# Neuroinflammation in schizophrenia: meta-analysis of *in vivo* microglial imaging studies

**DOI:** 10.1017/S0033291718003057

**Published:** 2018-10-25

**Authors:** Tiago Reis Marques, Abhishekh H Ashok, Toby Pillinger, Mattia Veronese, Federico E. Turkheimer, Paola Dazzan, Iris E.C. Sommer, Oliver D Howes

**Affiliations:** 1 Psychiatric Imaging Group, MRC Clinical Sciences Centre, Du Cane Road, London W12 0NN, UK; 2 Psychiatric Imaging Group, London Institute of Medical Sciences (LMS), Faculty of Medicine, Imperial College London, Du Cane Road, London W12 0NN, UK; 3 Department of Psychosis Studies, Institute of Psychiatry, Psychology & Neuroscience, Kings College London, London, UK; 4 Department of Neuroimaging, Institute of Psychiatry, Psychology & Neuroscience, Kings College London, London, UK; 5 Department of Psychiatry, Brain Center Rudolf Magnus, University Medical Center Utrecht, Utrecht, The Netherlands

**Keywords:** Microglia, neuroinflammation, schizophrenia, TSPO

## Abstract

**Background:**

Converging lines of evidence implicate an important role for the immune system in schizophrenia. Microglia are the resident immune cells of the central nervous system and have many functions including neuroinflammation, axonal guidance and neurotrophic support. We aimed to provide a quantitative review of *in vivo* PET imaging studies of microglia activation in patients with schizophrenia compared with healthy controls.

**Methods:**

Demographic, clinical and imaging measures were extracted from each study and meta-analysis was conducted using a random-effects model (Hedge's *g*). The difference in 18-kDa translocator protein (TSPO) binding between patients with schizophrenia and healthy controls, as quantified by either binding potential (BP) or volume of distribution (*V*_T_), was used as the main outcome. Sub-analysis and sensitivity analysis were carried out to investigate the effects of genotype, ligand and illness stage.

**Results:**

In total, 12 studies comprising 190 patients with schizophrenia and 200 healthy controls met inclusion criteria. There was a significant elevation in tracer binding in schizophrenia patients relative to controls when BP was used as an outcome measure, (Hedge's *g* = 0.31; *p* = 0.03) but no significant differences when *V*_T_ was used (Hedge's *g* = −0.22; *p* = 0.29).

**Conclusions:**

In conclusion, there is evidence for moderate elevations in TSPO tracer binding in grey matter relative to other brain tissue in schizophrenia when using BP as an outcome measure, but no difference when VT is the outcome measure. We discuss the relevance of these findings as well as the methodological issues that may underlie the contrasting difference between these outcomes.

## Introduction

Converging lines of genetic, epidemiological and clinical evidence indicate that inflammatory pathways are altered in schizophrenia. Over 20 epidemiological studies show that people with a history of infection or autoimmune diseases have an increased risk of schizophrenia (Benros *et al*., 2011, 2014; Khandaker *et al*., 2012, 2013; Miller *et al*., 2013). Variants in genes of the immune pathways have also been associated with an increased risk of schizophrenia (Stefansson *et al*., 2009). Moreover, the largest genome-wide association study in schizophrenia to date found a highly significant association between risk of schizophrenia (Schizophrenia Working Group of the Psychiatric Genomics, 2014) and a locus linked to the major immunohistocompatibility complex with subsequent work implicating microglial and complement activation in this pathway (Sekar *et al*., 2016). Similarly, elevated levels of a number of immune markers have been observed in schizophrenia (Tourjman *et al*., 2013). Studies have repeatedly (although not invariably) shown that patients with schizophrenia have increased serum concentrations of pro-inflammatory cytokines, including IL-1*β*, IL-6 and TNF-*α* (Upthegrove *et al*., 2014; Dickerson *et al*., 2016). Meta-analyses show that these are elevated in medication-naïve first-episode patients (Upthegrove *et al*., 2014) and in later stages of illness (Dickerson *et al*., 2016), with large effect sizes (e.g. Hedge's *g* > 2.2 for IL-6 and >1.1 for IL-1*β*) (Upthegrove *et al*., 2014). Moreover, studies of cerebrospinal fluid (CSF) have shown increased levels of pro-inflammatory markers in schizophrenia patients when compared with healthy controls, including IL-1*β*, IL-6 and S100B (Schmitt *et al*., 2005; Soderlund *et al*., 2009; Sasayama *et al*., 2013; Schwieler *et al*., 2015). While there is some evidence for increased inflammatory markers in blood and CSF in schizophrenia, this cannot be taken to suggest neuro-inflammation. The evidence for increased cerebral activation of the immune system is scarce. Post-mortem studies have demonstrated elevated markers for microglia, and morphological changes indicating microglial activation in schizophrenia patients (Bayer *et al*., 1999; Radewicz *et al*., 2000; Trepanier *et al*., 2016) although this was not seen in all brain regions (Steiner *et al*., 2006) and there are still a large number of null studies (Trepanier *et al*., 2016). However, a recent meta-analysis of post-mortem studies by van Kesteren *et al.* confirmed overall increased microglia density in schizophrenia, together with increased concentrations of pro-inflammatory proteins (van Kesteren *et al*., 2017).

Microglia are the resident immune cells of the central nervous system and act as major mediators of neuroinflammation. In the healthy brain, microglia retain a ‘quiescent’ phenotype where processes extend through the local environment to detect context-specific changes. In this stage, microglia cells produce neurotrophic factors, provide axonal guidance and regulate local cell proliferation. However, in response to inflammatory stimuli, the cells become activated, undergoing morphological changes and releasing pro-inflammatory cytokines. A number of risk factors for schizophrenia, notably pre-natal infection and psychosocial stress, are known to induce microglial activation (Juckel *et al*., 2011; Calcia *et al*., 2016). When microglia are activated, it increases the expression of the 18-kDa translocator protein (TSPO) (Cosenza-Nashat *et al*., 2009). TSPO can be measured *in vivo* with positron emission tomography (PET) radiotracers and so far a number of PET studies have investigated microglia activation in schizophrenia-spectrum disorders. However, findings have been inconsistent and so far they have only been partially reviewed quantitatively (Plaven-Sigray *et al*., 2018). We therefore aimed to synthesize PET imaging findings of microglial activation in patients with schizophrenia-spectrum disorders and healthy controls, and to discuss the implications of these findings in relation to both the pathophysiology of the disorder and drug development efforts.

## Methods

### Data source and study selection

**Fig. 1.**
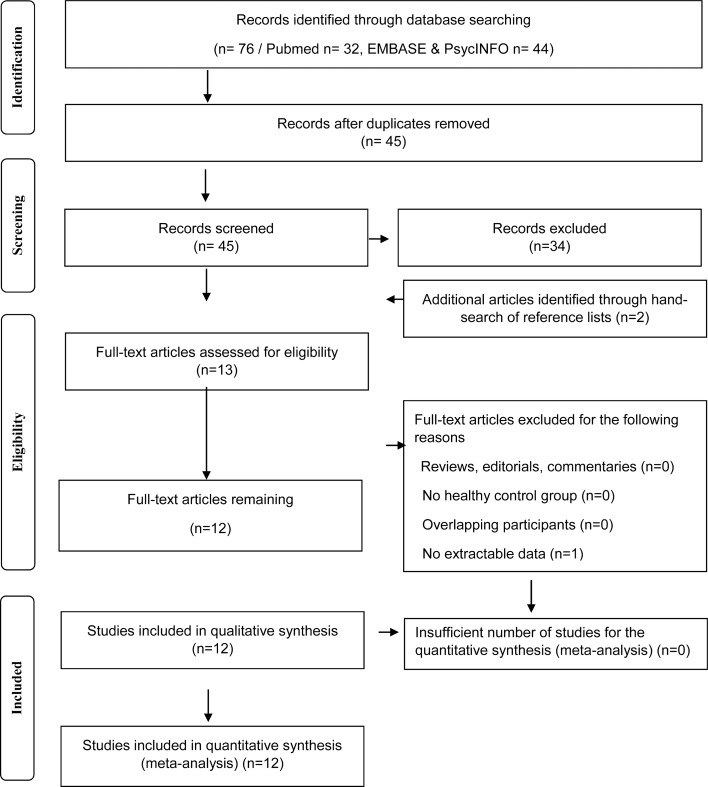
Flowchart showing the inclusion of studies for the meta-analysis.

The entire PubMed, EMBASE and PsycINFO databases were searched to identify manuscripts published from inception date until 12 January 2018. To be included in the meta-analysis, a study needed to report *in vivo* TSPO PET imaging data in patients with schizophrenia-spectrum disorders and in a healthy control group. All studies needed to report the mean and standard deviations for both groups (see Fig. 1).

### Data extraction

The main outcome measure was the difference in the TSPO imaging index between patients with schizophrenia-spectrum disorders and healthy controls. For all studies, we extracted the following variables: authors, year of publication, subject characteristics for the patient and healthy control group (group size, age, sex, diagnosis, duration of illness, antipsychotic medication, psychopathology rating scale scores), imaging characteristics (method, radiotracer) and modelling method. The estimation of pooled standard deviation was performed using the statstodo software (http://statstodo.com/ComMeans_Pgm.php). In order to extract data from studies where data were available only in a plot format, we have used the plot digitiser software (http://plotdigitizer.sourceforge.net/).

### Data analysis

The main outcome measure was the effect size determined using the TSPO tracer measure and quantified by either BP_ND_, BP_−P_ or *V*_T_ in the total grey matter in patients with schizophrenia-spectrum disorders and healthy controls using a random-effects model. A minimum of three studies were required to run a meta-analysis. The group mean and error measures were not reported by Banati and Hickie (2009), and although we requested the data from the authors, we were unable to obtain them. Bloomfield *et al*. (2016*a*, 2016*b*) reported the data in both *V*_T_ and distribution volume ratio (DVR) (using a multivariate analysis approach, where *V*_T_ in whole brain or cerebellum was used, along with age and TSPO genotype, as covariates in an analysis of covariance producing a marginal mean). The DVR method used in this study is not equivalent to the measures used in the other studies. Also Ottoy *et al*. (2018) reported *V*_T_ values only accounting for the vascular component (2TCM-1K) (Ottoy *et al*., 2018). In view of this, we requested the data for this meta-analysis and both authors provided the *V*_T_ values not accounting for the vascular component, determined in the same way as other studies (i.e. not covaried for age and gender). Therefore, the main analysis of *V*_T_ values included six studies using the 2TCM model.

A genetic variant at rs6971 in the TSPO gene, causing a non-conservative amino acid substitution, has been found to affect the binding of some TSPO PET tracers (Owen *et al*., 2012). Subjects who are homozygotes (LL) have low-affinity binding and have negligible TSPO binding *in vivo*. Those who are heterozygotes (HL) express both mixed affinity for TSPO (MAB), while those without the polymorphism (HH) have high-affinity binding (HAB) for TSPO (Guo *et al*., 2012). As on average MABs and HABs have a 22% difference in TSPO binding (Kreisl *et al*., 2013), we extracted the data for patients who are HABs and MABs separately to explore the effect of genotype in a sub-analysis.

Publication bias was assessed using visual inspection of funnel plots as well as regression test. Where potential publication bias was suspected, trim and fill analysis was conducted to correct for putatively missing studies. Heterogeneity was estimated using the *I*^2^ value (*I*^2^ values <50% indicate low-to-moderate heterogeneity, whereas *I*^2^ >50% indicate moderate-to-high heterogeneity). Leave-one-out sensitivity analyses were conducted to investigate the potential effect of an individual study on the outcome measure. A *p* value <0.05 (two-tailed) was taken as a significance level. The statistical analysis of the extracted data was conducted using the R statistical programming language version 3.2.2 with the ‘metafor’ package.

### Search strategy

The PubMed, EMBASE and PsycINFO databases were searched without language restrictions. The electronic search using EMBASE and PsycINFO were carried out together using Ovid. The following keywords were used: ‘(Positron Emission Tomography OR PET OR Single photon emission tomography OR SPET OR Single Photon Emission Computed Tomography OR SPECT) AND (schizophrenia OR schizophreniform OR psychosis) AND (microglia* OR microglia* activation OR TSPO OR Translocator protein OR peripheral benzodiazepine receptor OR peripheral benzodiazepine binding site)’. Review papers were also screened to search for additional studies.

### Inclusion and exclusion criteria

The inclusion criteria were: original studies in (1) patients with a diagnosis of schizophrenia or related psychotic diagnoses (including schizophreniform disorder; psychotic disorder not otherwise specified, brief psychosis), (2) reporting PET measures using a TSPO-specific ligand and (3) reporting data for the whole grey matter or grey matter regions. Studies that did not have a control group were excluded. Where there was sample overlap between studies, we included the largest one and excluded the other to avoid double counting.

### Outcome measures

The primary outcome measure was the effect size for the difference in TSPO PET measure in total grey matter between patients with schizophrenia-spectrum disorders and healthy controls. Where several studies only reported values for grey matter sub-regions, we averaged the grey matter regions to estimate the value for the whole grey matter. The PET studies predominantly reported the outcome either as binding potential (BP) or volume of distribution (*V*_T_). As these give different information, we conducted separate meta-analyses of these outcomes. The studies that used BP as an outcome measure have used either values obtained using microparameters from Simplified Reference Tissue Model (Holmes *et al*., 2016; van der Doef *et al*., 2016; Di Biase *et al*., 2017), but van Berckel *et al*. (2008) and Doorduin *et al*. (2009) have calculated BP using microparameters derived from 2TCM model. Despite this difference, the results are comparable as reviewed in PET receptor imaging consensus (Innis *et al*., 2007).

## Results

### TSPO binding

There were a total of 12 studies measuring TSPO tracer binding in 190 patients with schizophrenia-spectrum disorders and 200 healthy controls. Five of these studies were in chronic patients, seven in patients within the first 5 years of diagnosis, and two studies also included subjects at ultra-high risk for psychosis. See Table 1 for a summary of sample and study method characteristics. The rationale behind individual study exclusion is documented in supplementary information.

**Table 1.** Subject and methodological characteristics of the *in vivo* imaging studies of TSPO binding in schizophrenia compared with healthy controls (BP = 6; *V*_T_ = 6)

Author Year	Patients/controls (n)	Patient age mean (SD) years	Diagnosis	Duration of illness mean (SD) years	Current antipsychotic treatment?	Region(s) of interest (ROI)	Tracer	Genotyping	Outcome measures	Results in patients compared with controls
van Berckel *et al*. (2008)	10/10	24 (2)	Recent-onset schizophrenia (<5 years of illness)	3.1 (1.7)	Yes	Total grey matter	[^11^C]-PK11195	No	BP_−P_	↑
Doorduin *et al*. (2009)	7/8	31.2 (7.2)	Recent-onset schizophrenia (<5 years of illness)/psychotic disorder not otherwise specified	5.4 (5.4)	Yes	Total grey matter Frontal Cx/occipital Cx/temporal Cx/parietal Cx/basal ganglia/thalamus/hippocampus/midbrain/cerebellum/pons	[^11^C]-PK11195	No	BP	↑ (Hippocampus) ↔ (total grey matter and all ROI)
Takano *et al*., 2010	14/14	43.8 (7.4)	Chronic Schizophrenia	18.8 (12.2)	Yes	Total cortical regions Medial frontal cortex, dorsolateral frontal cortex, medial temporal cortex, lateral temporal cortex, parietal cortex, occipital cortex, thalamus, striatum, cerebellum, anterior cingulate cortex and posterior cingulate cortex	[^11^C]-DAA1106	No	BP_ND_	↔ (All regions)
Kenk *et al*. (2015)	18/27	42.6 (14.1)	Chronic schizophrenia	14.8 (8.8)	Yes	Hippocampus/mPFC Cx/temporal Cx/DLPFC Cx/V striatum/corpus callosum/cingulum/SLF/PLIC	[^18^F]-FEPPA	Yes	*V*_T_	↔ (All regions)
Bloomfield *et al*. (2016*a*, 2016*b*)	14/14	24.3 (5.4)	Ultra high risk for psychosis	N/A	No	Total grey matter/frontal Cx/temporal/cerebellum/brain stem	[^11^C]-PBR28	Yes	DVR *V*_T_	DVR: ↑ (total grey matter, frontal Cx, temporal Cx); ↔ (all other regions) *V*_T_: ↔ (all regions)
	14/14	47.0 (9.3)	Chronic schizophrenia	Not specified	Yes	Total grey matter/frontal Cx/temporal Cx/cerebellum/brain stem				DVR: ↑ (total grey matter, frontal Cx, temporal Cx); ↔ (all other regions) *V*_T_: ↔ (all regions)
Hafizi *et al*. (2016)	19/20	27.5 (6.7)	First-episode psychosis: schizophreniform disorder/delusional disorder/schizophrenia/psychosis NOS	2.8 (3.3)	No	Whole brain Total grey matter DLPFC/mPFC/Temporal Cx/hippocampus	[^18^F]-FEPPA	Yes	*V*_T_ DVR	↔ (All regions)
van der Doef *et al*. (2016)	19/17	26 (4)	First-episode psychosis (<5 years of illness)	1.3 (1.1)	Yes	Total grey matter Frontal Cx/temporal Cx/parietal Cx/striatum/thalamus	[^11^C]-PK11195	No	BP_ND_	↔ (All regions)
Coughlin *et al*. (2016)	12/14	24.1 (3.1)	Recent-onset schizophrenia (<5 years of illness)	2.2 (1.4)	Yes	Total grey matter Insula Cx/cingulate Cx/parietal Cx/frontal Cx/temporal Cx/occipital Cx/hippocampus/amygdala	[^11^C]- DPA-713	Yes	V_T_	↔ (All regions)
Holmes *et al*. (2016)	8/16	33 (9)	Schizophrenia	15 (7)	Yes	DLPFC/VLPFC/OFC/anterior cingulate Cx/parietal Cx	[^11^C]-PK11195	No	BP_ND_	↑ DLPFC, VLPFC and ACC ↔ (all other regions)
	8/16 (Shared control group)		Schizophrenia	4 (2)	No					↔ (all other regions)
Collste *et al*. (2017)	16/16	28.5 (8.4)	First-episode psychosis: schizophrenia/schizophreniform psychosis/psychosis NOS/brief psychosis	7.9 (9.6)	No	Total grey matter White matter/frontal Cx/temporal Cx/hippocampus	[^11^C]-PBR28	Yes	*V*_T_	↓ (Total grey matter, frontal Cx, temporal Cx, hippocampus) ↔ (white matter)
Di Biase *et al*. (2017)	18/15	20.6 (5.5)	Recent-onset schizophrenia	1.5 (1.0)	Yes	Dorsal frontal Cx/orbital frontal Cx/anterior cingulate Cx/medial temporal Cx/thalamus/insular Cx	[^11^C]-PK11195	No	BP_ND_	↔ (All regions)
	15/12	35.2 (6.6)	Chronic schizophrenia	13.6 (8.8)	Yes	Dorsal frontal Cx/orbital frontal Cx/anterior cingulate Cx/medial temporal Cx/thalamus/insular Cx	[^11^C]-PK11195	No	BP_ND_	↔ (All regions)
Ottoy *et al*., (2018)	11/17	30.0 (7.0)	Chronic schizophrenia	N/A	Yes	Cortical grey matter/cortical white matter/cerebellum/brainstem/thalamus/basal ganglia/hippocampus/amygdala	[^18^F]-PBR111	Yes	*V*_T_	↔ (All regions)

#### Studies reporting outcome as BP

**Fig. 2.**
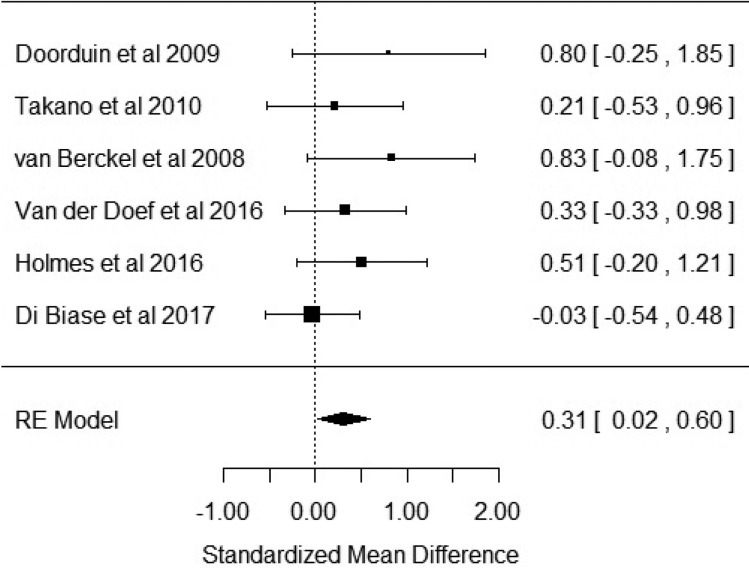
Forest plot showing the effect sizes for *in vivo* microglia measures in schizophrenia patients compared with controls as measured by translocator protein binding potential (BP) in total grey matter. There was a significant elevation in schizophrenia with an effect size = 0.31 (*p* = 0.03).

Six studies reported outcome measures as BP. Our results showed that BP was significantly elevated in patients with schizophrenia when compared with healthy controls with an effect size of 0.31 [Hedge's *g* = 0.31; *z* = 2.1; *p* = 0.03; 95% confidence interval (CI) 0.02–0.6] (Fig. 2). The *I*^2^ test revealed low heterogeneity (*I*^2^ = 0.58%; 95% CI 0–79%). Visual inspection of the funnel plot suggested asymmetry (online Supplementary Fig. S1). The regression test was significant (*t* = 4.5; df = 4; *p* = 0.01). The trim and fill analysis showed three putatively missing studies on the left side. The results were not significant after correcting for these studies (Hedge's *g* = 0.13; *z* = 0.96; *p* = 0.34; 95% CI −0.14 to 0.4). The results were significant in two out of six in the leave-one-out analysis, with effect sizes varying from 0.27 to 0.47. Five out of the six studies used the [^11^C]-PK11195 ligand, with the sub-analysis of these studies revealing a significant elevation of BP in patients with schizophrenia with an effect size of 0.35 (CI 0.01–0.7; *p* = 0.046) (online Supplementary Fig. S2).

#### Studies reporting the outcome as volume of distribution (*V*_T_)

**Fig. 3.**
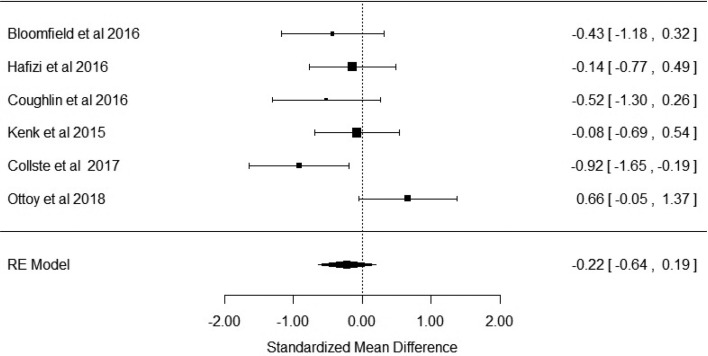
Forest plot showing effect sizes for *in vivo* microglia measures in schizophrenia patients compared with controls as measured by volume of distribution of translocator radiotracer (*V*_T_) in total grey matter. There were no significant changes in patients compared with controls (effect size = −0.22, *p* = 0.296).

Six studies reported the outcome measure as *V*_T_. Figure 3 shows that there was no difference in *V*_T_ in patients with schizophrenia when compared with healthy controls (Hedge's *g* = −0.22; *p* = 0.296; CI −0.64 to 0.19). The *I*^2^ test revealed moderate–high heterogeneity (*I*^2^ = 53%; 95% CI 0–92%). Visual inspection of funnel plot suggested asymmetry (online Supplementary Fig. S3). However, the regression test was not significant (*t* = −0.73; df = 4; *p* = 0.5). Trim and fill analysis showed two missing studies in the right side. The effect sizes varied from −0.08 to −0.37 in the leave-one-out analysis. We extracted data for high- (HABs) and mixed-affinity binders (MABs) separately from these studies to conduct a sub-analysis stratified by genotype. Bloomfield *et al*. reported only one patient who was a MAB, precluding accurate estimation of the effect size. Thus, this study was not included in the sub-analysis of MAB subjects (Bloomfield *et al*., 2016*b*). There was no significant difference between patients and controls in the high-affinity binder sub-analyses (effect size −0.27; *p* = 0.19; CI −0.68 to 0.13). However, there was a significant difference in the MAB (effect size −0.56; *p* = 0.03; CI −1.08 to −0.03).

## Discussion

Our main findings are that TSPO PET tracer binding is significantly elevated in patients with schizophrenia relative to controls when BP is used as an outcome measure, with a small-to-moderate effect size (Hedge's *g* = 0.31; *p* = 0.03), but there is no significant difference when the tracer volume of distribution (*V*_T_) is used as the outcome measure (Hedge's *g* = −0.22; *p* = 0.296). In the following section, we consider methodological factors and the implications of our findings.

### Methodological considerations

We identified potential publication bias for both outcome measures, and in the case of BP meta-analysis, the results were no longer significant when adjusted for putative missing studies, which if present could potentially affect our findings. Heterogeneity was low for those studies that used BP as an outcome measure and moderate to high in those that used *V*_T_ as an outcome measure. For this meta-analysis, we used a random-effects model, which is robust to between-study variations. We acknowledge that four of the 12 studies used in this meta-analysis analysed patients with schizophreniform disorder, psychotic disorder not otherwise specified, and brief psychosis as well as patients with schizophrenia (Doorduin *et al*., 2009; van der Doef *et al*., 2016; Collste *et al*., 2017). Two of the studies do not provide a break-down of individual patient participant diagnoses, other than to classify patients as presenting with first-episode psychosis (van der Doef *et al*., 2016; Hafizi *et al*., 2017), thus up to 52/190 (27%) of patients included in the meta-analysis are defined as presenting with a schizophreniform disorder, rather than a definitive diagnosis of schizophrenia. Therefore, our results could be influenced by the inclusion of other psychotic disorders, although the low heterogeneity observed for the meta-analysis using BP as an outcome measure at least suggest that including individuals with broader psychotic diagnoses alongside schizophrenia did not have a major impact on the results. Five out of the six studies included in the BP meta-analysis used the first-generation tracer [^11^C]-PK11195, whereas the *V*_T_ studies used second-generation tracers. Thus, the difference between BP and *V*_T_ outcomes could reflect tracer differences, as [^11^C]-PK11195 is known to have low brain penetration and high non-specific binding, which is a significant limitation of this tracer relative to the second-generation tracers (Fujita *et al*., 2008). However, although the outcome of the BP study that used a different tracer ([^11^C]-DAA1106) is negative, the results using this tracer are not an outlier, suggesting findings may not entirely be accounted for by tracer differences. It has been suggested that TSPO expression may change during the course of the disorder, which could account for the differences in findings between studies (Notter *et al*., 2018). However, a recent study by Di Biase *et al.* using BP as an outcome measure showed no differences between at-risk mental state individuals, recent onset schizophrenia and chronic schizophrenia (Di Biase *et al*., 2017), and our BP and *V*_T_ meta-analyses both included studies of chronic and recent onset illness, suggesting that this does not clearly explain the differences between our BP and *V*_T_ findings. Ultimately, longitudinal studies in patients are required to determine if there are changes during the course of the illness. Other differences between the studies using BP and *V*_T_, such as differences in tracer and modelling approaches, could contribute to the differences between our BP and *V*_T_ meta-analytic findings. Another potential issue is that not all studies accounted for a genetic variant at rs6971 in the TSPO gene. Six out of the 12 studies included in this meta-analysis did not report genotyping (van Berckel *et al*., 2008; Doorduin *et al*., 2009; Takano *et al*., 2010; Holmes *et al*., 2016; van der Doef *et al*., 2016; Di Biase *et al*., 2017). Out of these six studies, five used the [^11^C]-PK11195 tracer while one study used the tracer [^11^C]-DAA1106. However, genotyping has been shown not to be necessary in studies using [^11^C]-PK11195, as *in vitro* studies have shown that this tracer binds to a different site on the TSPO to the locus affected by the rs6971 variant and, consequently, there is no difference in affinity to TSPO between high-affinity and low-affinity binders (Owen *et al*., 2012). However, our sub-analyses for the other tracers stratified by genotype showed group differences only remained significant in the MAB groups. We caution against overinterpretation of this finding given the small sample, but suggest it warrants further investigation. Another potential methodological limitation is that a number of studies did not account for partial volume effects. However, as brain volumes tend to be reduced in schizophrenia, partial volume effects would not explain the elevation in BP and, if anything, would tend to reduce the effect size, which means our results may underestimate the true effect. Additionally, in those cases where total grey matter TSPO levels were not available, we have averaged the grey matter regions to estimate the value for the whole grey matter. Although this is a common procedure in PET meta-analyses (Howes *et al*., 2012; Kambeitz *et al*., 2014; Ashok *et al*., 2017), it can constitute a potential limitation of this study. Finally, most of the studies in this meta-analysis included patients who were being treated with antipsychotics. Preclinical studies *in vitro* have found antipsychotic treatment to reduce microglial activation (Zheng *et al*., 2008), although recent *in vivo* work has found an increase after olanzapine, but a reduction with risperidone (Zhu *et al*., 2014; Cotel *et al*., 2015; Crum *et al*., 2016). Critically, none of these studies measured TSPO levels, so it remains unknown if antipsychotics alter TSPO expression. Interestingly, both the studies by Holmes *et al.* and Di Biase *et al.* showed that unmedicated patients have lower BP when compared with antipsychotic-treated patients and healthy controls (Holmes *et al*., 2016; Di Biase *et al*., 2017). However, this analysis was based only on data provided by 12 patients and further work is thus needed to understand whether antipsychotic treatment could have affected our findings.

### Interpretation of findings

The non-displaceable BP (BP_ND_) measures the tracer binding in the tissue of interest relative to another brain region selected to have negligible specific binding, to give specific binding in the region of interest (Mintun *et al*., 1984). An issue for the measurement of BP_ND_ is that there is no brain region with negligible TSPO expression, and consequently no ideal reference region for TSPO tracers (Turkheimer *et al*., 2015; Bloomfield *et al*., 2016*a*; Narendran and Frankle, 2016). Thus the elevation in BP we report could reflect an increase in specific binding and/or a reduction in non-specific binding in grey matter relative to the reference brain tissue. In contrast, the volume of distribution (*V*_T_) measures the total amount of tracer in the brain region relative to that in the blood (Innis *et al*., 2007). Volume of distribution is generally the preferred method of quantification of PET tracers where there is no reference region but, critically for group comparisons, assumes blood tracer binding is unaltered between groups (Innis *et al*., 2007). However, TSPO tracers may bind to a number of sites in the blood, including acute phase and inflammatory plasma proteins, such as *α*1-acid glycoprotein (AGP) that are known to be elevated in schizophrenia (Telford *et al*., 2012; Tourjman *et al*., 2013). As TSPO tracers bind to AGP (Lockhart *et al*., 2003), there is the potential for a systematic bias between patients and controls that could affect the quantification of *V*_T_, and potentially mask an elevation in the patients’ brain as compared with controls. There is inconsistency in findings in schizophrenia, with one study showing elevation in the plasma concentration levels of [^11^C]-PBR28 in patients with schizophrenia relative to controls (Bloomfield *et al*., 2016*a*), while two others using [^18^F]-FEPPA and [^11^C]-PBR28 show no differences in plasma concentration levels (Hafizi *et al*., 2016; Collste *et al*., 2017). There is also evidence for reduced levels of binding of TSPO tracers to TSPO (previously known as the peripheral benzodiazepine receptor) on platelets in schizophrenia, with reductions of ~30% reported in some studies (Gavish *et al*., 1986; Weizman *et al*., 1986), although potentially only in certain sub-types of schizophrenia (Wodarz *et al*., 1998). Thus, it is possible that changes in either plasma protein binding and/or platelet binding could systematically affect *V*_T_ values in the disorder, but it should be recognized that there is no direct evidence that plasma binding alters *V*_T_ values in humans (Cumming *et al*., 2018). Importantly, in a recent study where protein binding was measured, no significant group differences between drug-naive first-episode psychosis patients and healthy controls were observed, suggesting that *V*_T_ changes are not explained by an effect of protein binding (Collste *et al*., 2017). Further studies are thus needed to clarify if there is an impact of this on the measurement of *V*_T_ in schizophrenia. Nevertheless, while this is a potential concern for studies using *V*_T_ as the outcome measure, blood binding should not affect the studies that use a ratio approach, as these methods report tracer uptake relative to another brain region rather than blood. Furthermore, TSPO is expressed on the endothelial cells of brain blood vessels as well as on the outer layer of mitochondria in microglia (Rizzo *et al*., 2014), and both can be accounted for in the PET analysis (Turkheimer *et al*., 2015). Few of the studies included in this meta-analysis have accounted for endothelial binding. In view of this, in our meta-analysis, we have included the results not accounting for this compartment. A separate meta-analysis was conducted including the data accounting for the endothelial binding and the results were very similar suggesting this is unlikely to be a major influence on our findings.

Taken together, our meta-analytic findings suggest an elevation in TSPO tracer binding in total grey matter relative to other brain tissue, but not relative to blood, with the caveat that the relative increase is largely based on studies using PK11195, which has a lower specific signal. Thus, this could reflect an increase in TSPO in grey matter or a reduction in TSPO in the reference region, or altered non-specific binding in the brain (Cumming *et al*., 2018). It should also be noted that reductions in TSPO have been reported in some pro-inflammatory states (Narayan *et al*., 2017). Finally, TSPO may also be expressed on astrocytes (Cosenza-Nashat *et al*., 2009). While altered TSPO expression on astrocytes may contribute to the differences, the post-mortem findings of unaltered astrocytic but elevated microglial markers (Trepanier *et al*., 2016), including elevated TSPO binding (Kreisl *et al*., 2013), are more suggestive of a microglia activity increase in schizophrenia. However, until further work has been conducted to determine if changes in translocator protein expression in schizophrenia are specific to microglia, conclusions about the specificity of changes to microglia should be treated cautiously.

### Implications of our findings and future directions

Our different findings depending on the outcome measure used point towards the existence of potential methodological problems in TSPO imaging, raising questions over the interpretation of the elevation in grey matter TSPO binding relative to a reference region in patients with schizophrenia when compared with healthy controls. A recent individual participant data meta-analysis of second-generation radioligand studies, with *V*_T_ as the outcome measure, showed a reduction in *V*_T_ in patients relative to healthy controls (Plaven-Sigray *et al*., 2018). Although these results seem to be in contrast to the absence of differences in *V*_T_ in our meta-analysis, our meta-analysis included one more second-generation study showing no differences in *V*_T_ between schizophrenia patients and healthy controls (Ottoy *et al*., 2018), which may justify the differences in results between meta-analysis. Ultimately, the definitive test of the importance of TSPO and/or microglia activation in schizophrenia will be to pharmacologically target them with a selective drug or monoclonal antibody combined with PET imaging to confirm target engagement and evaluate the relationship between change in microglial activation and symptomatic improvement. Secondly, it remains to be determined if microglia activity is altered across the different stages of the disorder. Epidemiological and preclinical evidence which indicates that neuroinflammation *in utero* and early development may predispose to schizophrenia (Meyer, 2013). However, the PET results have been inconsistent, with one study using the tracer [11C]-PBR28 showing an elevation in relative TSPO binding in people at ultra high risk of psychosis using the ratio but not with the *V*_T_ approach (Bloomfield *et al*., 2016b), while two studies using *V*_T_ as the outcome measure, using the tracer [18F]-FEPPA and [11C]-PK11195, showed no differences in TSPO binding between healthy controls and in individuals at ultra high risk of psychosis for psychosis (Hafizi *et al*., 2016; Di Biase *et al*., 2017). Unfortunately, there were not sufficient studies to proceed with a sub-analysis of studies conducted in the early phase of illness. Future research should focus on at-risk mental state individuals and early in the development of the illness and use longitudinal designs to determine the role of microglial activity in the different stages of the disorder. Ideally, these studies should be conducted before antipsychotic medication initiation, as it would also help to clarify the potential role of antipsychotics in microglia activity. Interestingly, it has been shown that pro-inflammatory cytokines are elevated in first-episode patients who do not respond to antipsychotic treatment relative to those who do respond (Mondelli *et al*., 2015), which suggests that increases in microglia activity may be specific to a sub-group of patients, consistent with neurobiological sub-types of schizophrenia (Howes and Kapur, 2014). Studies that focus on patients that do not respond to conventional antipsychotic medication should be able to shed light on this topic. When interpreting our results is also important to note that this meta-analysis focused on total grey matter TSPO binding, and there was insufficient data for a meta-analysis of specific brain regions. Although TSPO is ubiquitous and expressed across the whole brain, we cannot exclude that microglia activation in schizophrenia is differentially expressed in specific regions within the brain. Indeed, early studies by Doorduin *et al*. (2009) and van Berckel *et al*. (2008) suggest increased TSPO binding in the medial temporal cortex, and Bloomfield *et al*. (2016*b*) using the DVR approach also found evidence of this in people at ultra high risk of psychosis. Finally, future studies are needed to address the methodological issues and sources of variance discussed above. This could be potentially achieved by obtaining individual patient data from each individual study and applying both ratio and volume of distribution models to determine if PET modelling or study differences drive the opposing BP and volume of distribution findings. In addition, we recommend that new studies present results for both modelling approaches so that consistency of findings can be compared and support future meta-analysis, while the role of vascular binding in PET TSPO quantification should be clarified.

## Conclusion

In conclusion, there is evidence for a moderate effect size elevation in TSPO tracer binding in grey matter in schizophrenia-spectrum disorders when using BP as an outcome measure, but no changes when *V*_T_ is the outcome measure used. These results suggest that potential methodological differences between TSPO studies need to be accounted for and addressed in future studies and keep open the discussion over the existence of an increase in microglia activity in patients with schizophrenia-spectrum disorders.

## Supplementary material

For supplementary material accompanying this paper visit https://doi.org/10.1017/S0033291718003057. click here to view supplementary material
