# Supplementary material for: Neuroinflammation in schizophrenia: meta-analysis of in vivo microglial imaging studies
Source: Psychol Med. 2018 Oct 25;49(13):2186–96. doi: 10.1017/S0033291718003057 (PMC6366560; doi:10.1017/S0033291718003057)
Supplement: Supplementary file 1 [file S0033291718003057sup001.docx]

**Supplementary Material**

**Supplementary figure 1: Funnel plot of studies reporting outcome measures with BP method**


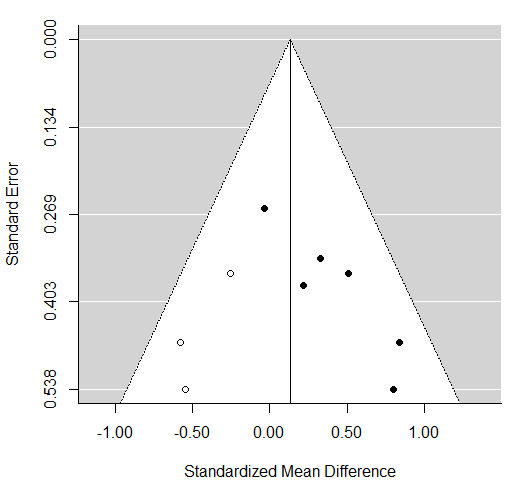


**Supplementary figure 2: Forest plot of studies which used [^11^C]-PK11195 ligand**

**
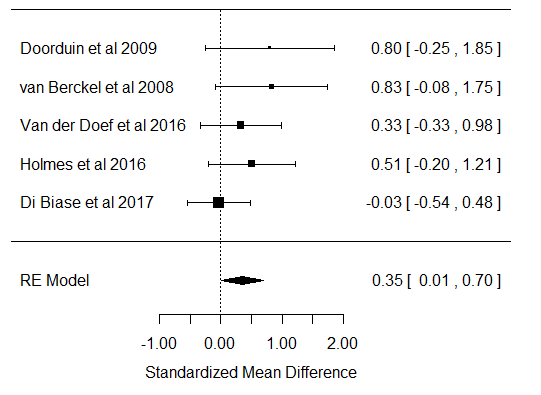
**

**Supplementary figure 3: Funnel plot of studies reporting outcome measures with V_T_ method**

**
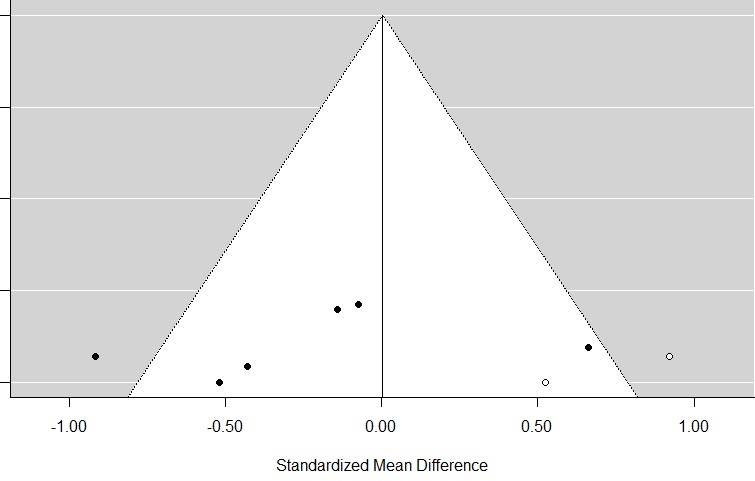
**

**Supplementary figure 4: Forest plot of studies reporting outcome measures with the V_T_ method in mixed affinity binders**

**
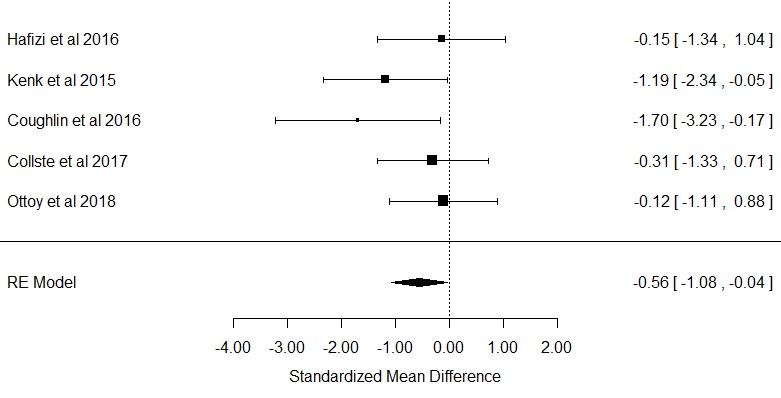
**

**Supplementary figure 5: Forest plot of studies reporting outcome measures with the V_T_ method in high affinity binders**

**
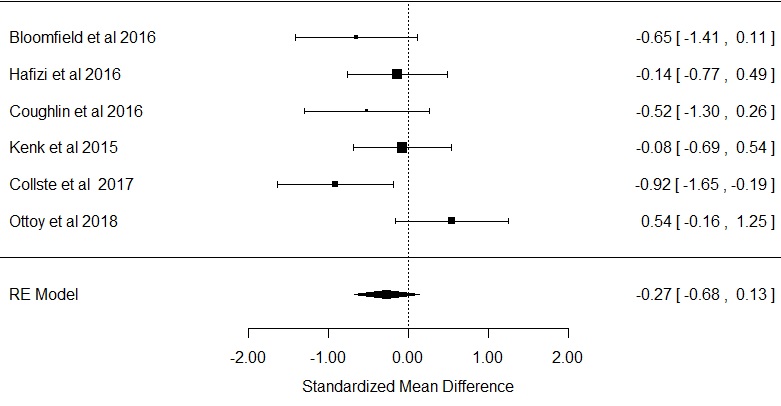
**
